# Adjusting time-of-day and depth of fishing provides an economically viable solution to seabird bycatch in an albacore tuna longline fishery

**DOI:** 10.1038/s41598-023-29616-7

**Published:** 2023-02-14

**Authors:** Eric Gilman, Tom Evans, Iain Pollard, Milani Chaloupka

**Affiliations:** 1 Fisheries Research Group, The Safina Center, Honolulu, USA; 2 Key Traceability, Portsmouth, UK; 3 Ecological Modelling Services Pty Ltd and Marine Spatial Ecology Lab, University of Queensland, Brisbane, Australia

**Keywords:** Ecology, Conservation biology, Environmental impact

## Abstract

Marine megafauna exposed to fisheries bycatch belong to some of the most threatened taxonomic groups and include apex and mesopredators that contribute to ecosystem regulation. Fisheries bycatch is a major threat to the conservation of albatrosses, large petrels and other pelagic seabirds. Using data sourced from a fisheries electronic monitoring system, we assessed the effects of the time-of-day and relative depth of fishing on seabird and target species catch rates for a Pacific Ocean pelagic longline fishery that targets albacore tuna with an apparently high albatross bycatch rate. Using a Bayesian inference workflow with a spatially-explicit generalized additive mixed model for albacore tuna and generalized linear mixed regression models both for combined albatrosses and combined seabirds, we found that time-of-day and fishing depth did not significantly affect the target species catch rate while night-time deep setting had > 99% lower albatross and total seabird catch rates compared to both deep and shallow partial day-time sets. This provides the first evidence that night-time setting in combination with fishing deep reduces seabird catch risk and may be commercially viable in this and similar albacore tuna longline fisheries. Findings support evidence-informed interventions to reduce the mortality of threatened seabird bycatch species in pelagic longline fisheries.

## Introduction

Marine megafauna captured as incidental bycatch in fisheries belong to some of the most threatened taxonomic groups and include apex and mesopredators that have essential contributions towards regulating ecosystem structure, functions and stability^1–3^. Bycatch is a major threat to the conservation of pelagic seabirds, in particular for albatrosses and large petrels^4–6^.

Effective methods to avoid and minimize catch rates and remediate the risk of fishing mortality of threatened bycatch species are now available for some gear types and some threatened taxa^7,8^. However, there has been mixed progress in their uptake, in part, due to costs to commercial viability (economic viability, practicality and crew safety) as well as due to weak enabling environments of government management frameworks and market-based mechanisms^9–13^.

Albacore tuna (*Thunnus alalunga*) longline fisheries typically set either deep during the day with relatively high risk to seabirds mainly at higher latitudes, or shallow at night with high risk to threatened epipelagic species such as marine turtles and silky (*Carcharhinus falciformis*) and oceanic whitetip sharks (*C. longimanus*)^12,14–19^. Determining the economic viability of pelagic longline deep, night setting to target albacore tuna in temperate zones is a priority.

To identify evidence-informed seabird bycatch management interventions for a pelagic longline fishery that targets albacore tuna across temperate latitudes of the north and south Pacific Ocean, this study analyzed data obtained from a fisheries electronic monitoring (EM) system. The objectives of analyses were to assess the effect of relative depth and the time-of-day of fishing on seabird and target species catch rates. These operational factors are also informative predictors of marine turtle and species-specific elasmobranch catch and at-vessel mortality rates^8,15,18,20^. Findings support evidence-informed interventions to reduce the mortality of threatened seabird species in albacore tuna longline fisheries.

## Results

All but one of 611 observed captured seabirds were retrieved dead. Gear setting occurred for a mean of 5.9 h (95% CI: ± 0.03 h). Seabirds had a nominal catch rate of 0.384 per 1000 hooks, and 70% of captured seabirds were albatrosses. The EM analyst was unable to identify any of the captured seabirds to the species level.

Of 1,029 sets in the full study sample, 74% (764) were conducted in areas where seabird bycatch mitigation methods are required by the Western and Central Pacific Fisheries Commission (WCPFC) and the Inter-American Tropical Tuna Commission (IATTC), which are tuna-regional fisheries management organizations. Of these 764 sets, 13% met definitions of night setting. These 764 sets began an average of 7.4 h after nautical dusk, and all but one of the sets began after nautical dusk. Thus, all but one of the sets that did not meet night setting definitions was due to the time of the end of the set being past nautical dawn^21^ or local sunrise^22^. Of these sets, 23% exceeded nautical dawn or local sunrise by < 1 h, and 64% by < 3 h.

**Figure 1:**
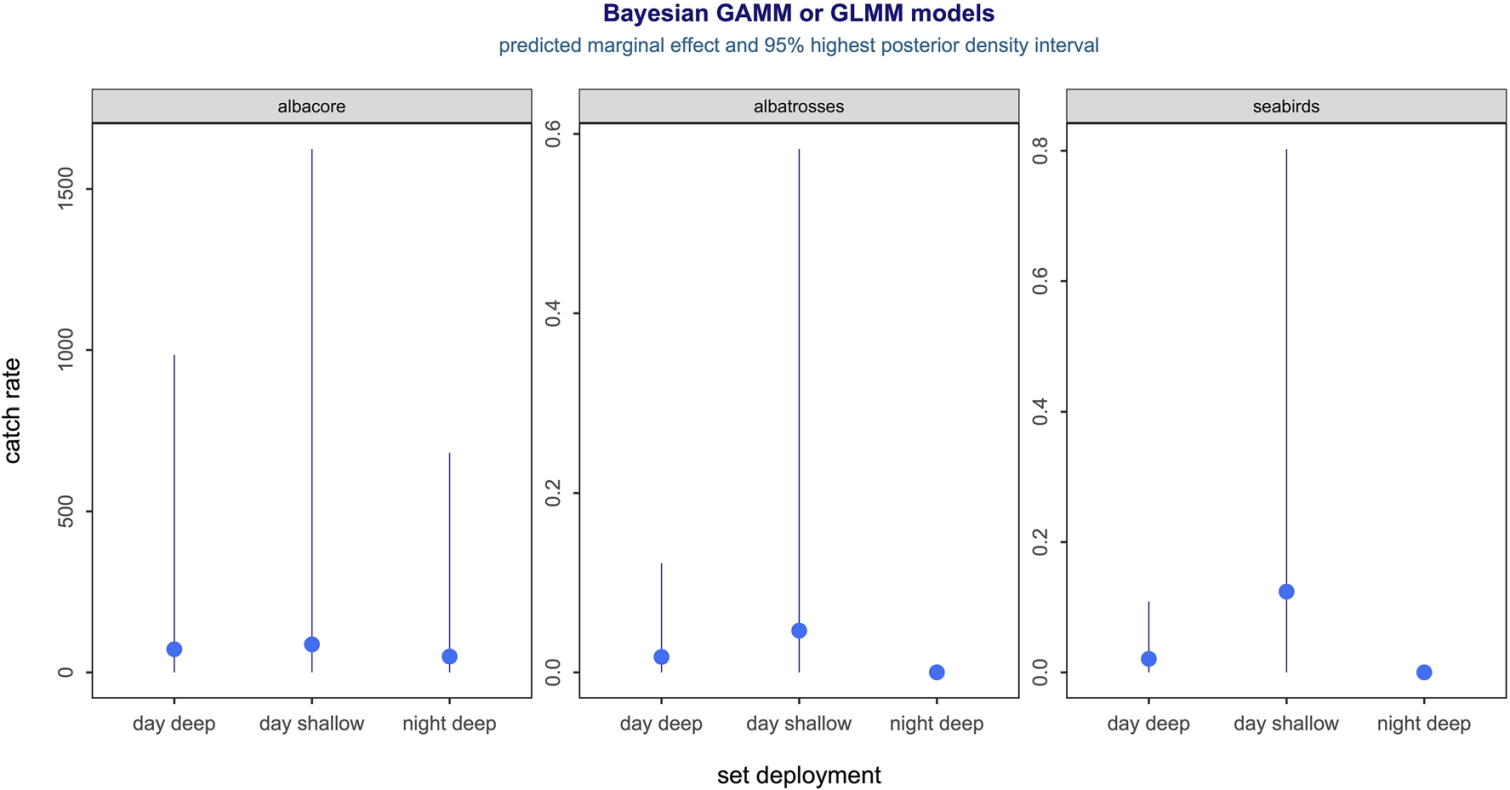
Predicted 3-category set deployment treatment effect for albacore catch per set (left), probability of catching at least 1 albatross per set (middle), and probability of catching at least 1 seabird per set (right). The albatross night-deep 95% highest posterior density interval (HDI, 0 to 0.0003) and seabird night-deep 95% HDI (0 to 0.0004) are concealed by the solid dot. Solid dot = median predicted marginal effect. Vertical bar = 95% HDI.

Figure 1 presents the predicted marginal treatment effects of compliance with night setting definitions and hooks between floats for albacore tuna, combined albatrosses and combined seabird species, averaged over the sampling period. There was no significant difference in predicted median albacore tuna catch rates (expected catch per set) between the three set categories. The median albacore tuna catch rate in day-shallow sets was higher than the rate of the other two set categories, but this effect was not significant, with only a 58% probability that there was an effect. The predicted median albatross catch rate (expected probability of catching at least 1 albatross per set) was significantly lower on night-deep sets compared to the other two set categories, with > 99% probability of an effect. We can only be > 85% sure that the albatross catch rate was higher in day-shallow sets compared to day-deep sets, and this effect was not significant. As with albatrosses, the predicted median catch rate for combined seabird species (expected probability of catching at least 1 seabird per set) was significantly lower in night-deep sets compared to the other two set categories, with > 99% probability of this effect. The night-deep predicted median albatross and seabird catch rates were > 99% lower than both the day-deep and day-shallow rates (Fig. 1). We can also be > 95% sure that the seabird catch rate was higher in day-shallow sets compared to day-deep sets. The day-deep median seabird catch rate was 83% lower than the day-shallow rate.

## Discussion and conclusions

The estimated seabird bycatch rate in this fishery of 0.384 per 1000 hooks, with an albatross catch rate of 0.9 per set, is high relative to other tuna longline fisheries^4,23–25^ and a concern given the threatened conservation status of albatrosses and petrels that are exposed to the fishery. Albatrosses and petrels are two of the three most threatened groups of seabirds^5^. Of the 29 albatross and large petrel species listed under the Agreement on the Conservation of Albatrosses and Petrels, 19 are categorized as threatened^6,26^.

All but 1 of the 611 observed captured seabirds was retrieved dead, suggesting that in this fishery seabirds are captured during the set and drown during the gear soak^10^. The largest seabird conservation gain can therefore be achieved by reducing seabird capture risk during setting as opposed to during the gear retrieval. The EM analyst did not identify any seabird captures to the species level, preventing an assessment of species-specific risks. Previous assessments of the same EM system^27,28^ found that simple modifications to the EM system would enable substantial improvements to species identification and other data quality improvements.

Having sets end earlier in the day so that they are completed prior to nautical dawn within the WCPFC convention area and prior to local dawn in the IATTC convention area would enable the vessels to reduce seabird catch risk. To avoid reducing the set duration, this could be implemented in combination with initiating sets earlier (but still after nautical dusk in the WCPFC convention area and more than one hour after local sunset in the IATTC convention area)^21,22^. Additional research is needed to assess the costs to economic viability and practicality of this proposed shift to earlier times of day of the start and end of sets. Compliance with night setting can be determined through electronic monitoring systems, by conventional onboard observers, as well as through satellite-based systems (e.g., vessel monitoring systems or VMS and automatic identification system or AIS). Night setting thus provides a broader range of compliance monitoring options relative to some other seabird bycatch mitigation methods such as whether crew deploy hooks outside of the prop wash, dye bait or attach weights within a specified distance from hooks^9^.

The significantly lower catch rates of combined albatrosses and combined seabird species in WCPFC- and IATTC-defined night sets is consistent with an extensive body of studies finding lower seabird catch rates during night sets than day-time sets because most seabirds that are susceptible to pelagic longline bycatch do not forage at night^12,29^. However, night setting can result in higher catch rates of crepuscular and nocturnal foraging seabird species in fisheries that overlap with these species (e.g., northern fulmars *Fulmarus glacialis*,^30^).

The number of pelagic longline hooks that are attached between two floats, used here to define three categories of sets (see the Methods section), is an approximate index for *relative* but not *absolute* fishing depth. The more hooks that are deployed between two floats, the deeper the depth range of the hooks along a catenary curve will be if all other variables are constant^31,32^. The number of hooks between floats, however, is a poor predictor of actual fishing depth. This is because variability in several other factors that affect fishing depth, including shoaling from ocean currents and wind, and variability in other gear designs (e.g., length of mainline between floats, mainline diameter, distance between floats, distance between the point of attachment to the mainline of the first branchline and the point of attachment of the nearest floatline, distance between branchlines, and length of branchlines and floatlines) affect the absolute depth range of the hooks^31,32^.

The higher catch rate of non-albatross seabird species in shallower sets with *ca.* 11 hooks between floats (Fig. 1) may have been a result of a larger proportion of hooks being accessible to deep-diving seabird species, such as shearwaters and some petrel species, during setting, such as if hooks in shallower sets are more likely to be accessible to seabirds further astern. Only one of the four vessels made shallower sets. There may have been differences in additional informative predictors of seabird catchability between the shallower-setting and deeper-setting vessels. For example, the density of seabirds attending the vessels during setting, location where crew deploy baited hooks (e.g., into or away from the prop wash), offal management practices and wind speed^33^ could have explained the observed differences in seabird catch rates between shallower vs. deeper setting vessels. But these variables were not possible to explore with this limited EM dataset. Additional research is needed to determine the mechanism causing this observed effect of relative fishing depth on seabird catch rates.

The lack of a significant difference in albacore tuna catch rates between the three set categories suggests that it may be commercially viable to conduct night-time deep sets in the temperate Pacific in fisheries that conduct relatively long gear soaks, as is the practice in this fishery. Additional research is required to determine the economic effects from changes in catch rates of *all* principal market species. Shifting the time-of-day of setting to meet night setting definitions would eliminate some of the gear soak during the day-time. This might reduce the vertical overlap between the fishing gear and some pelagic apex predator principal market species whose diel vertical migration cycles mirror movements of their prey, which occurs with albacore tuna in some regions^15,34–36^. Depending on the vertical diel distribution pattern of a particular species, the time of day of gear setting and haulback in combination with the fishing depth can significantly affect catch rates^15^. Large changes in the time-of-day and depth of fishing in pelagic longline fisheries employing short gear soaks might cause larger changes in catch rates of principal market species relative to fisheries with long gear soaks. Additional research is needed to assess the effects on the economic viability and practicality of switching from deep daytime to deep night setting, in particular for small-scale pelagic longline fisheries that have relatively short gear soak durations.

Having pelagic longline vessels switch from shallower partial day-time to deeper night setting may result in multispecies conflicts. While this could decrease the catch risk of primarily diurnal foraging seabird species, and would reduce catch risk of threatened epipelagic species such as silky and oceanic whitetip sharks and marine turtles, it would increase catch rates of nocturnal foraging seabird species and threatened mesopelagic species such as thresher sharks^15,18,30^. Bycatch management strategy evaluation^37^ could enable identifying and accounting for these conflicts so that unavoidable tradeoffs are intentional and best meet objectives for multispecies bycatch management.

To identify evidence-informed bycatch management interventions for a data-limited albacore tuna longline fishery, this study analyzed a short time series of EM data. Night-time deep setting did not significantly affect the target species catch rate and had a > 99% lower seabird catch rate compared to both deep and shallow partial-day sets. Night-time deep setting, which avoids diurnal foraging seabird species as well as threatened epipelagic sharks and marine turtles, may be commercially viable in this and similar albacore tuna longline fisheries.

## Methods

EM data were collected on four pelagic longline vessels with an overall (maximum) hull length of 53 m. EM equipment and reviewing software were produced by the company Satlink. The EM system included four video cameras with fields of view of: the deck at the: setting station at the stern, processing deck, outboard side of the rail at the fish door off the hauling station, and outboard side of the rail astern of the hauling station. During 15 trips made by the four vessels, 3.4 million hooks were deployed during 1,029 sets made between 28 May 2018 and 9 December 2020, at fishing grounds that ranged from 37.7^o^N to 39.0^o^S and from 148.8^o^E to 91.5^o^W. The vessels used a hydraulic mainline line shooter to set the mainline slack, used forage fish species (Pacific saury *Cololabis saira* and sardines, Clupeidae) for bait and used a single hook type (4.6 cm-wide, 10° offset circle hooks). Vessels deploy *ca*. 3,300 hooks per set, gear setting occurs for *ca.* 6 h, and the gear maximum soak duration (the time between the beginning of the set and end of the haul) was *ca.* 21 h.

The effect of the time-of-day of setting and relative fishing depth on combined albatross species, combined seabird species, and albacore tuna catch rates was assessed for 656 sets that occurred in locations where WCPFC or IATTC require the employment of seabird bycatch mitigation measures^21,22^. (Of the 1,029 sets in the full study sample, 764 sets were conducted in areas where seabird bycatch mitigation methods are required by WCPFC and IATTC. Of these, EM analysts reviewed 656 sets to record captures of albacore tuna and seabirds). The 656 sets were deployed during 10 trips made by 4 albacore tuna longline vessels undertaken during 2019 and 2020 at fishing grounds in the following two areas on the high seas of the Pacific Ocean: (1) 23$^\circ$N to 38$^\circ$N, 147$^\circ$E to 146$^\circ$W; and (2) 39$^\circ$S to 30$^\circ$S, 176$^\circ$W to 114$^\circ$W. Sets were categorized as either:

1. Night, deep sets: Sets that met definitions of night setting, with a mean of 21.5 hooks between floats (range 20–22 hooks);
2. Not night, deep sets: Sets not meeting night setting definitions, with a mean of 21.0 hooks between floats (range 19–22 hooks), and
3. Not night, shallow sets: Sets not meeting night setting definitions with a mean of 11.4 hooks between floats (range 10–12 hooks).

For convenience we refer to “not-night” as simply “day”. Table 1 summarizes the sample sizes for the three categories of set types, hooks per set category, and number of captures of albacore tuna, combined albatrosses (not identified to the species level by the EM analyst) and other non-albatross seabird species within each set type, also not identified to the species level by the EM analyst. WCPFC defines night setting as not setting between nautical dawn (begins when the sun is 12 degrees below the horizon in the morning) and nautical dusk (begins when the sun is 6 degrees below the horizon in the evening), while IATTC’s definition is not setting between local sunrise (when the sun first breaks the horizon) and one hour after local sunset^21,22^.

**Table 1:** Summary of sample sizes of three categories of set types and albacore tuna and seabird catch.

Set category	No. sets	No. hooks	No. albacore tuna	No. albatrosses	No. other seabirds
Night-deep	91	298,813	1,746	0	0
Day- shallow	183	675,738	3,811	17	114
Day-deep	382	1,241,835	6,122	17	0

The set was the sampling unit for this assessment. There were 11,679 albacore tuna caught in the dataset with a simple mean catch of *ca.* 17 albacore tuna per set although 472 sets (*ca.* 72%) recorded zero albacore catch. On the other hand, albatross and other seabird catch in the sets was rare—for instance only 34 albatrosses were caught in 17 sets while 148 seabirds of all species (including the albatrosses) were caught in 33 sets.

The statistical modelling approach was based on a Bayesian inference workflow^38,39^ based on fitting (1) a spatially explicit GAMM or geoGAMM^43^ that accounted for the georeferenced location of the start of each set for albacore tuna as there was adequate data for this species to do so, and (2) generalized linear mixed regression models (GLMMs) for albatrosses and seabirds^40^ with an appropriate response-specific likelihood for each of the 3 species being: inflated negative likelihood for the albacore tuna catch model^42,45^, and Bernoulli likelihood for both the catch model for combined albatross species and the catch model for combined seabird species (including albatrosses) (see^43^ for more details).

Seabird bycatch was a rare event in this fishery and so the response metric for both the combined albatross species and combined seabird species models was a binary metric (for example: 0 = no catch on a set, 1 = at least one catch on a set) and are sampled from a Bernoulli probability distribution and appropriately modelled using a regression model with Bernoulli likelihood—which is a special case of a binomial likelihood but now with a single trial^46^.

All Bayesian geoGAMMs and GLMMs were fitted to the species-specific bycatch conditional on the 3-category set deployment treatment effect (day-deep, day-shallow, night-deep) while accounting for potentially informative predictors such as sampling year (2019, 2020), season within sampling year and spatial effects. Fishing effort was accounted for as an *offset(log(hooks))* term. Group-level or random effect structures (intercepts-only) included in the GAMM and GLMMs were the identity of the 10 trips and the identity of the 4 vessels sampled from a multivariate Gaussian distribution^47^ to account for any correlated or trip- and/or vessel-specific heterogeneity in the catch rates not accounted for by the other predictors.

All models were fitted using the Stan computation engine^48^ via the brms interface^49^ and implemented using weakly informative regularizing priors^50^. Model fit was assessed using procedures and metrics proposed by Gabry et al.^38^ and Vehtari et al.^51^. For instance, leave-one-out cross-validation^52^ and Bayesian stacking^41^ were used to evaluate the relative expected predictive accuracy of the albacore geoGAMM with negative binomial likelihood compared to a geoGAMM with zero-inflated negative binomial likelihood.

We used the same procedures to evaluate the predictive accuracy for the following models to evaluate the comparative importance or not of including georeferenced spatial and seasonality effects on tuna catch rate: (1) a GLMM without either spatial or seasonal effects, (2) a geoGAMM with both spatial and seasonal effects, and (3) a geoGAMM without seasonal effects. The best fit model was a GAMM with spatial effects but not seasonality (model 3) with the weight of evidence strongly in favour of this model > 93%—this model was then used for further inference. There were too few albatrosses and seabirds to address more elaborate models that could account for the location and seasonality of sets as conducted for albacore tuna.

The posterior samples for all models were sourced from 4 Markov chains and 5,000 iterations per chain after a warmup of 1,000 iterations per chain. Therefore, the posterior for each estimate comprised 20,000 samples or draws that were then used to derive the species-specific treatment effects and uncertainty intervals (HDIs or highest posterior density intervals:^53^). The effect summaries sourced from each model were then adjusted for variable sample size of the treatment effect using the predicted marginal means approach^54^ and derived using the posterior predictions from the models using the emmeans package for R^55^. These predicted or estimated marginal effects for set deployment with 95% HDIs are summarized in Fig. 1. All inference is then based on these predicted marginal effects.

In any experimental setting it is important to be able to conclude that there was an effect when there really was an effect. And it is equally as important to be able to conclude that there was no effect when there was no effect. This can be done here using indices of existence and significance in a Bayesian setting^44^. A probability statement about the *existence* of a particular effect and its direction, set-deployment effects, can be determined with those 20,000 draws using the probability of direction metric proposed recently by Makowski et al.^44^—also known as the maximum probability of an effect.

## Data Availability

The third-party data underlying this study are available upon reasonable request from the owner through dataset custodian Key Traceability, who can be contacted at fisheriesresearchgroup@gmail.com.
